# Utility of Serum Biomarkers of Myocardial Fibrosis in High-Gradient Severe Aortic Stenosis: An Explorative Cardiovascular Magnetic Resonance Imaging-Based Study

**DOI:** 10.3390/diagnostics15091143

**Published:** 2025-04-30

**Authors:** Megan R. Rajah, Erna Marais, Gerald J. Maarman, Emma Doubell, Anton F. Doubell, Philip G. Herbst

**Affiliations:** 1 Division of Cardiology, Department of Medicine, Faculty of Medicine and Health Sciences, Stellenbosch University and Tygerberg Hospital, Cape Town 8000, South Africa; 2 Centre for Cardio-Metabolic Research in Africa, Division of Medical Physiology, Faculty of Medicine and Health Sciences, Stellenbosch University, Cape Town 8000, South Africa; et4@sun.ac.za (E.M.);; 3 Department of Medicine, Faculty of Medicine and Health Sciences, Stellenbosch University, Cape Town 8000, South Africa; 16091485@sun.ac.za

**Keywords:** myocardial fibrosis, biomarkers, transforming growth factor-beta 1, collagen synthesis, aortic stenosis, cardiovascular magnetic resonance imaging

## Abstract

**Background:** Myocardial fibrosis in aortic stenosis (AS) is associated with a significant risk of poor clinical outcomes. Myocardial fibrosis can be evaluated using cardiovascular magnetic resonance (CMR) imaging and may be useful for risk-stratifying patients at high risk for poorer outcomes. A circulating biomarker of fibrosis may be a cheaper, more accessible alternative to CMR in lower-to-middle-income countries. This study evaluated the correlation between serum biomarkers of myocardial fibrosis (TGF-β1, PICP, and PIIINP) with CMR markers of myocardial fibrosis (T1 mapping, extracellular volume fraction (ECV), and late gadolinium enhancement (LGE)). **Methods:** Twenty-one high-gradient (mean gradient ≥ 40 mmHg) severe AS (aortic valve area < 1.0 cm^2^) participants underwent T1 mapping and LGE imaging using CMR. Blood serum was collected for enzyme-linked immunosorbent assays of the listed biomarkers. **Results:** Serum TGF-β1 was associated significantly with the global T1 relaxation time on CMR (r = 0.46 with 95% CI 0.03 to 0.74, *p* = 0.04). In the high T1 time group (1056 vs. 1023 ms), trends toward elevated serum TGF-β1 concentration (13,044 vs. 10,341 pg/mL, *p* = 0.08) and ECV (26% vs. 24%, *p* = 0.07) were observed. The high T1 and trend towards elevated TGF-β1 concentration in this group tracked adverse LV remodeling and systolic dysfunction. There were no significant associations between PICP/PIIINP and T1 mapping or between the biomarkers and LGE quantity. **Conclusions:** Serum TGF-β1 is a potential surrogate for diffuse interstitial fibrosis measured by T1 mapping and ECV on CMR. Serum PICP and PIIINP may be less appropriate as surrogate markers of fibrosis in view of their temporal trends over the course of AS. Larger studies are needed to validate the utility of TGF-β1 as a marker of diffuse fibrosis and to evaluate the utility of serial PICP/PIIINP measurements to predict decompensation.

## 1. Introduction

Aortic stenosis (AS) is one of the most common valve diseases worldwide, with a dramatically increasing prevalence in several countries over the past two decades [1]. For both rheumatic heart disease and calcific degenerative AS, mortality rates and disability-adjusted life-years lost remain high despite advances in aortic valve intervention [1]. The only definitive therapy currently available is valve replacement by means of surgical aortic valve replacement or, more recently, using a transcatheter approach (transcatheter aortic valve implantation or TAVI) [2,3]. Although definitive, these interventions are reserved only for those who reach severe disease accompanied by the presence of symptoms or systolic impairment (left ventricular ejection fraction (LVEF) < 50%) [2,3,4]. Independent of the traditional parameters used to risk stratify patients for valve replacement, the status of the left ventricle (LV) in terms of remodeling and fibrotic burden have also been shown to independently predict outcomes in AS [5,6,7,8]. Since there is evidence of increased mortality risk in some groups that do not meet the current guideline criteria for valve replacement (e.g., in asymptomatic severe AS and some patients with moderate AS) [2,3,9,10], there is now a growing interest in incorporating additional predictive parameters into a new risk stratification strategy that has the potential to reclassify some high-risk patients for intervention.

Several parameters for improved risk stratification have been suggested. These include strain analysis of the LV and use of B-type natriuretic peptide (BNP)/N-terminal Pro-B-type natriuretic peptide (NT-pro BNP) biomarkers, both of which still require further validation in the context of AS [2,11]. Left ventricular myocardial fibrosis has also been suggested as an additional parameter for the risk stratification of patients with AS and may be used to prompt earlier decision-making for valve replacement [2,12,13,14]. Myocardial fibrosis in AS has been associated with adverse LV remodeling and has been suggested to coincide with the onset of symptoms and the transition to decompensation, i.e., systolic impairment, all of which are important determinants of poor outcomes in AS [5,6,14,15,16,17,18,19,20]. A direct association between myocardial fibrosis and poor outcomes has been shown, and this relationship appears to be dose-dependent [21,22,23,24,25].

Myocardial fibrosis in AS can be detected and quantified using endomyocardial biopsy or non-invasively using cardiovascular magnetic resonance (CMR) imaging [12,13,14]. Two tools are utilized for myocardial fibrosis assessment and quantification on CMR, including parametric T1 mapping for diffuse interstitial fibrosis and late gadolinium enhancement (LGE) imaging for replacement fibrosis [13,14]. While these techniques are well-validated and used widely in developed countries for a range of conditions, including AS, access to CMR in low-to-middle-income countries remains limited [26]. The development of a circulating biomarker for myocardial fibrosis has the potential to serve as a cheaper, more easily accessible alternative to CMR in low-to-middle-income countries and may be a useful tool for the early recognition of patients at high risk for poorer outcomes.

Markers of fibrosis regulation and metabolism have been suggested as potential candidates for circulating biomarkers of myocardial fibrosis [27]. These include transforming growth factor-beta 1 (TGF-β1) and its downstream regulatory effect on the synthesis of types I and III collagen. These represent the two predominant forms of collagen in the myocardial extracellular space whose synthesis has been shown to be dynamically dysregulated through different stages of several cardiovascular diseases, including systemic arterial hypertension and heart failure [27,28]. While these proteins have been investigated in animal models of pressure overload, human studies remain limited, and whether these proteins have potential utility as fibrosis biomarkers remains inconclusive, with calls for further studies [29,30,31,32,33]. This was an explorative study aimed to investigate the association between serum TGF-β1, the procollagen I carboxy-terminal propeptide (PICP), and the procollagen III amino-terminal propeptide (PIIINP) with myocardial fibrosis assessed by T1 mapping and LGE imaging on CMR.

## 2. Methods

### 2.1. Study Design

Participants were prospectively enrolled for the study at Tygerberg Hospital, Western Cape, South Africa, between March 2022 and September 2024. Participants meeting the European Society of Cardiology’s criteria [2] for high-gradient (≥40 mmHg) severe AS were included. These criteria were based on standard transthoracic echocardiography-derived aortic valve area (AVA < 1.0 cm^2^), mean transaortic pressure gradient (≥40 mmHg), and peak velocity (≥4.0 m/s). Participants with comorbid structural heart disease, including cardiac amyloidosis, those with other hemodynamically significant valvular lesions, those with significant coronary artery disease diagnosed on coronary angiography, and patients with inflammatory rheumatological and/or thyroid conditions were excluded from the study. All participants had a peripheral venous sample collected for biochemical analysis, and all participants underwent CMR imaging. An analysis of the cohort divided into two groups based on a normal (global T1 time < 1040 ms) or high T1 relaxation time (global T1 time ≥ 1040 ms) was also performed. This cutoff value was based on the T1 range for normal myocardium in our local population. Ethical approval was received from the Human Research Ethics Committee of Stellenbosch University (Reference: S21/11/251 PHD), and written informed consent was obtained from every participant.

### 2.2. CMR Image Acquisition

Cardiovascular magnetic resonance (CMR) imaging was performed on a Siemens Magnetom Aera 1.5 Tesla (Siemens, Erlangen, Germany) scanner using a standardized protocol developed in accordance with the Society of Cardiovascular Magnetic Resonance guidelines [34]. Cine images for a morphological, volumetric, and functional analysis were obtained using a breath-held, electrocardiogram-gated, balanced steady-state free precession sequence. Images were obtained in the standard cardiac views (two-chamber, three-chamber, four-chamber, and short-axis views). Typical cine image acquisition parameters included an 8 mm slice thickness with a 2 mm gap where applicable, 25 phases, TR. 35–45 ms, TE. 1.2 ms, matrix size 156 × 192 with voxel size ± 1.9 × 1.9 mm. Parametric T1 mapping images were obtained using a modified look-locker inversion recovery sequence 5(3)3 in the standard cardiac views. Typical image parameters included a slice thickness of 8 mm, matrix size 204 × 256, and voxel size ± 1.5 × 1.5 mm. Gadolinium-based contrast agent at a dose of 0.2 mmol/kg (Gadovist, Bayer Pharmaceuticals, Leverkusen, Germany) was administered for post-contrast LGE imaging and post-contrast T1 mapping. Late gadolinium enhancement images were acquired ±10–15 min after contrast administration using an inversion recovery gradient echo sequence. Typical image parameters included an 8 mm slice thickness with no gap where applicable, TR. 784–804 ms, TE. 1.20–1.89 ms, TI 260–280 ms, and matrix size 154 × 196. Post-contrast T1 mapping was repeated ±15 min after contrast administration for extracellular volume (ECV) fraction evaluation.

### 2.3. CMR Image Analysis

The CMR image analysis was performed using CVI42 (version 5.13.7, Circle Cardiovascular Imaging, Calgary, AB, Canada) software. The left ventricular end-diastolic volumes (LVEDV), end-systolic volumes (LVESV), stroke volumes (LVSV), masses (LVM), and ejection fractions (LVEF) were derived by contouring endo- and epicardial borders in end-diastole and end-systole on every slice of the short axis cine stack. In accordance with the consensus recommendations, trabeculations were included in the blood pool, and papillary muscles were manually excluded from the blood pool [35]. The volumes were indexed using the Mostellar-derived body surface area and are denoted by the letter “i” in this article. Meridional end-systolic wall stress (ESWS) was estimated non-invasively using the sum of systolic blood pressure and mean gradient as a surrogate for LV pressure in the validated equation described by Reicheck et al. [36]. The left ventricular global function index (LVGFI) was calculated using the equation validated and described by Mewton et al. [37], and the pattern of remodeling was assigned according to the classification system described by Dweck et al. [38]. The global native T1 and T2 relaxation times were derived by contouring endo- and epicardial borders on every slice of the short axis T1 and T2 mapping stacks. The ECV was estimated using the post-contrast T1 relaxation times, and the equation previously validated and published by Flett et al. [39]. Late gadolinium enhancement was quantified using a manual visual method as well as the signal threshold versus reference mean method at a threshold of three standard deviations above the mean signal intensity of remote myocardium [35].

### 2.4. Blood Sample Collection and Storage

A peripheral venous sample of ±12–15 mL was collected from the antecubital fossa of each participant. The samples were collected into serum separator tubes (BD, Johannesburg, South Africa) and left to clot for at least one hour. The samples were centrifuged at 1.1 relative centrifugal force for 10 min at room temperature (Eppendorf Centrifuge 5702 R, Dubai, United Arab Emirates). The resultant serum was collected and aliquoted for storage at –80 degrees Celsius until the time of analysis. To assess for spurious releases of TGF-β1 during the serum separation process, a platelet count was performed on blood collected into ethylenediaminetetraacetic acid-containing tubes (BD, Johannesburg, South Africa).

### 2.5. Enzyme-Linked Immunosorbent Assay (ELISA)

The serum concentrations of total TGF-β1, PICP, and PIIINP were determined using commercially available ELISA kits (TGF-β1: ELK Biotechnology, Wuhan, China, ELK 1185; PICP and PIIINP: Elabscience, Houston, TX, USA, E-EL-HE6030 and EL-H0183 respectively). Serum dilutions with assay buffer for TGF-β1 (two-step dilution of 1:10 followed by 1:100) and PICP (1:10) were performed in accordance with the manufacturer recommendations, bringing the diluted sample values within the linear ranges of the standard curves (62.5–1000 pg/mL and 23.4–1500 pg/mL respectively). No serum dilutions were required for PIIINP. The serum samples and standards were incubated in triplicate on pre-coated 96 well plates at 37 °C for 80–90 min. After the initial incubation, the plates were decanted and incubated with the relevant biotinylated antibody solutions at 37 °C for 50–60 min followed by streptavidin-horse radish peroxidase (HRP) solutions at 37 °C for 30–50 min, with wash steps in between. Finally, the plates were incubated in the dark with an HRP substrate reagent at 37 °C. After 15–20 min, the reactions were stopped, and the plates were read immediately on a microplate reader at a wavelength of 450 nm (FLUOstar Omega, version 6.20, BMG LabTech, Ortenberg, Germany). A standard curve was constructed using the standard well measurements, and the sample serum concentrations were calculated by interpolating the readings on the standard curve. For TGF-β1 and PICP, final concentrations were obtained through multiplication by their respective dilution factors.

### 2.6. Statistical Analysis

GraphPad Prism (version 10.0.2, GraphPad, Boston, MA, USA) was used for statistical analysis. The cohort of patients evaluated in this study forms part of a larger study, and this work was deemed explorative. The sample size calculation was, therefore, based on the detection of differences in left ventricular morphology, volumes, function, and tissue characteristics using CMR. The Shapiro-Wilk test was used for normality testing. Continuous variables were presented as mean ± standard deviation where normally distributed and as the median and interquartile range where non-normally distributed. Normally distributed data were compared using the unpaired *t*-test and non-normally distributed data was compared using the Mann-Whitney U test. A *p*-value < 0.05 was considered statistically significant. Correlation analysis for normally distributed data was performed using the Pearson method and for non-normally distributed data, with the Spearman method.

## 3. Results

### 3.1. Study Population

Twenty-one participants with high-gradient severe AS [AVA 0.65 ± 0.21 cm^2^ and mean gradient 50 (41–56) mmHg] were recruited for the study. The mean age of the population was 60 ± 11 years, with more than half the cohort being female [13 (62%)]. The baseline characteristics of the total population are described in Table 1. The most frequently experienced symptoms included dyspnoea followed by fatigue and then chest pain (Table 1). While the prevalence of concomitant hypertension was high in the total cohort (13 (62%)), the mean blood pressure was within clinically acceptable limits (128/75 ± 20/13 mmHg). The cohort was subdivided based on the global native T1 time. The baseline characteristics of each subgroup are described in Table 1. No significant differences in baseline characteristics between the two subgroups were found (Table 1).

### 3.2. Biomarkers in the Total Cohort

**Figure 1:**
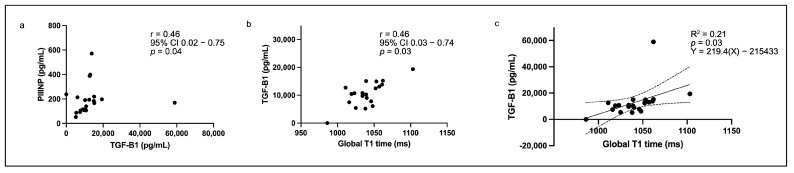
Scatter plot graphs showing significant associations between (**a**) transforming growth factor-beta 1 (TGF-β1) and procollagen III amino-terminal propeptide (PIIINP), (**b**) TGF-β1 and global native T1 relaxation time, and (**c**) simple linear regression for the prediction of TGF-β1 concentration using global T1 relaxation time.

In the total cohort (*n* = 21), the serum concentrations of TGF-β1, PICP, and PIIINP were 10,715 (7644–14,387) pg/mL, 235.8 (134.5–327.9) ng/mL and 169.7 (107.9–216.3) pg/mL, respectively. The serum concentration of TGF-β1 correlated poorly with the platelet count (r = 0.28, 95% confidence interval −0.19 to 0.64, *p* = 0.24). While a weak association was found between TGF-β1 and PICP (r = −0.07, 95% confidence interval −0.50 to 0.38, *p* = 0.75), a significant moderate association was observed between TGF-β1 and PIIINP (r = 0.46, 95% confidence interval 0.02 to 0.75, *p* = 0.04) (Figure 1a). A significant moderate association between TGF-β1 and global T1 relaxation time was also found (r = 0.46, 95% confidence interval 0.03 to 0.74, *p* = 0.04) (Figure 1b,c). There were no significant associations between TGF-β1 and ECV or LGE mass. Similarly, there were no significant associations found between PICP/PIIINP and global T1 relaxation time, ECV, or LGE mass.

### 3.3. Left Ventricular Remodelling and Serum TGF-β1 in the Subgroup with High Global T1 Relaxation Time

**Figure 2:**
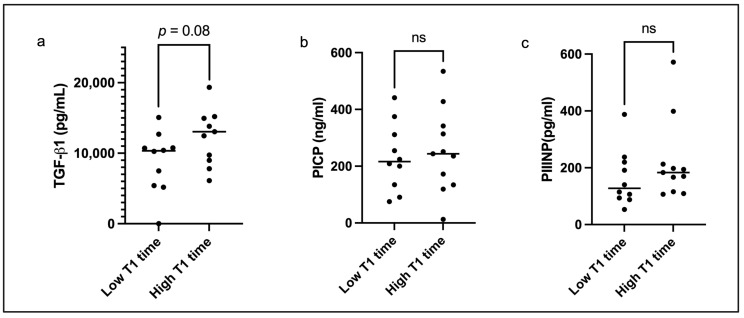
Graphs illustrating differences in serum concentrations of (**a**) transforming growth factor-beta 1 (TGF-β1), (**b**) procollagen I carboxy-terminal propeptide (PICP), and (**c**) procollagen III amino-terminal propeptide (PIIINP) between patients with a low global T1 relaxation time (1023 ± 16 ms) compared to those with a high T1 relaxation time (1056 ± 18 ms). The letters “ns” represents non-significant differences where *p* > 0.1.

The results of an analysis of the population divided into two groups based on the global native T1 relaxation time are shown in Table 2. The mean T1 relaxation time in the low T1 group was 1023 ± 16 ms compared to 1056 ± 18 ms in the high T1 group (*p* < 0.01). A trend towards higher serum TGF-β1 concentration was observed in the high T1 group [13,044 (8999–15,192) vs. 10,341 (5340–11,246), *p* = 0.08] (Figure 2a). No significant differences in the PICP or PIIINP concentrations were found between the two groups (Figure 2b,c). In those with a high global T1 relaxation time, worse LV remodeling was observed with significant cavity dilation (LVEDVi 116.8 ± 39.5 vs. 83.2 ± 29.6 mL/m^2^) and significant LV hypertrophy (LVMi 91.0 ± 22.8 vs. 67.0 ± 16.9 g/m^2^) (Table 2). A normal remodeling pattern was observed in the low T1 group, while a decompensated phenotype predominated in those with a high T1 relaxation time (Table 2). Significant systolic impairment was observed in the high T1 relaxation group [LVEF 30 (21–56) vs. 55 (38–69)%] with a significantly decreased LVGFI (23.4 ± 10.7 vs. 33.9 ± 9.0). There was a trend towards a higher ECV in the high T1 group (Table 2) and no significant differences in LGE mass between the two groups.

## 4. Discussion

This explorative study relating serum biomarkers to myocardial fibrosis on CMR found a significant association between TGF-β1 and global T1 relaxation time with a trend towards higher TGF-β1 concentrations in those with high T1 relaxation times. These findings suggest that there is a potential role for TGF-β1 as a surrogate marker of diffuse interstitial fibrosis, but further larger studies are needed. There were no significant associations between PICP/PIIINP and T1 mapping and no associations between TGF-β1, PICP, or PIIINP and replacement fibrosis measured and quantified on LGE imaging.

### 4.1. TGF-β1 Is a Potential Surrogate Marker of Diffuse Interstitial Fibrosis

In the absence of myocardial edema and/or infiltration, an abnormally high T1 relaxation time and/or ECV on CMR suggests the presence of diffuse interstitial myocardial fibrosis [40]. Transforming growth factor beta-1 is considered a key role player in the development of myocardial fibrosis [33,41,42,43], and in this study, a significant association between serum TGF-β1 and T1 relaxation time was observed in the total cohort. In the subgroup with an abnormally high T1 relaxation time for our magnetic field (normal reference ranges published previously by our group) [44], a trend towards higher ECV and serum TGF-β1 concentration was found, suggesting the potential role of this biomarker as a surrogate for diffuse interstitial fibrosis measured by T1 on CMR. This finding is promising, given strong evidence that T1 and ECV are powerful independent predictors of mortality and other clinical outcomes in AS [5,6,18].

In addition to the strong predictive utility of T1 mapping and ECV on mortality in AS, their association with adverse LV remodeling and decompensation has also been shown and is further supported by the findings of our study [5,17,19,20]. Treibel et al. showed that worse patterns of LV remodeling are associated with higher T1 relaxation times and ECV [19]. Similarly, those with high T1 times in our cohort displayed concentric hypertrophy or decompensated patterns of LV remodeling with systolic impairment compared to a normal phenotypic pattern with preserved systolic function in those with normal T1 times. Adverse patterns of remodeling (concentric hypertrophy, concentric remodeling, and LV dilation, in particular) have been shown in several studies to predict mortality, even in conservatively managed patients with severe asymptomatic AS and moderate AS [7,8,45,46]. Not only has an increased T1 time been associated with worse LV remodeling but also with subclinical systolic dysfunction [20]. This highlights the potential role of T1 mapping and, perhaps, serum TGF-β1, which appears to track T1 time, as early markers of the transition towards adverse remodeling and systolic dysfunction. This may prove clinically useful in identifying patients who could benefit from earlier intervention, but further larger studies are needed to confirm this.

### 4.2. No Biomarker Associations with Replacement Fibrosis

The serum concentrations of TGF-β1, PICP, and PIIINP were not associated with replacement fibrosis as measured on LGE imaging. While the small sample size may have contributed to this observation, other explanations were considered. The quantification of replacement fibrosis using LGE imaging in AS is fraught with challenges [47]. More specifically, there is no consensus on the optimal post-processing method to use in AS, and biopsy-validated studies remain rare [25,35,47,48,49,50]. Additionally, the coexistence of diffuse interstitial fibrosis, together with the presence of small non-bright scars (and microscars, which are likely missed on LGE imaging), pose a challenge to the precision of the various post-processing techniques used for LGE quantification [47]. In this study, two different methods (a manual visual method and a semi-automated three standard deviation signal threshold versus reference mean method) were chosen for LGE quantification based on the best available evidence [47,48]. Neither method yielded a significant association with the serum concentrations of these biomarkers. Whether this was related to the technical difficulties faced with LGE quantification in the context of AS or other reasons is unknown.

A biological explanation for this observation also exists. Traditionally, large areas of cardiomyocyte loss have been suggested to underlie the development of replacement fibrosis [51]. The mechanisms underlying the development of replacement fibrosis in AS, however, are not well understood. A supply-demand perfusion mismatch with consequent ischaemic-related injury and cell death has been suggested as one mechanism [52,53]. Still, large cardiomyocyte losses, as seen in myocardial infarction, are not considered likely. There is evidence to suggest that diffuse interstitial fibrosis precedes the development of replacement fibrosis in AS [51,54], and whether replacement fibrosis in AS simply reflects areas of pronounced accumulation of diffuse interstitial fibrosis over time is unknown. If diffuse interstitial fibrosis is the preceding event and, therefore, the event activated by TGF-β1 signaling, it is then understandable that TGF-β1 would correlate better with T1 mapping rather than LGE quantity on CMR. LGE may, therefore, track the chronicity of disease better at the end of the spectrum (low gradient severe AS). This patient cohort was, however, not included in this study.

### 4.3. Lack of Association Between PICP/PIIINP and Myocardial Fibrosis on CMR

Types I and III collagen are the predominant collagens found in the myocardial extracellular space [27,28]. In another study of patients with AS, a gradual decline in collagen III synthesis coupled with an increase in collagen I synthesis was observed in patients deteriorating towards systolic impairment [31]. In other words, the synthesis of these proteins in AS is not a static process but rather dynamic and likely influenced by a variety of factors. This switch from type III to type I collagen synthesis is not likely to be reflected by changes in T1 relaxation time, which simply measures the accumulation of fibrosis and not the specific type of collagen being deposited. Since patients present at varying (unknown) time points in the trajectory of their disease, evaluation of these biomarkers at a single time point may introduce significant heterogeneity in the measurements relative to T1 time, and this could explain the lack of significant results observed in this cohort.

While the temporality of PICP and PIIINP may render them less useful as surrogate markers of T1 relaxation time in AS, this temporality may be clinically useful in other ways. For example, patients with severe asymptomatic disease and preserved systolic function are likely to be managed conservatively with a “watch and wait” approach. Serial measurements of PICP and PIIINP over the watch and wait period may be useful in predicting a transition to decompensation before decompensation ensues by waiting for a fall in PIIINP concentration with a simultaneous rise in PICP concentration. This could enable clinicians to intervene early, before patients develop clinical determinants of poor outcomes such as systolic dysfunction.

## 5. Limitations

This study was limited by a small sample size, therefore, limiting the power to potentially show statistically significant differences in the biomarkers between patients with a normal versus high global T1 relaxation time on CMR. The larger cohort from which this study’s population emanated was not designed to include a normal control arm of patients without AS, thus, limiting the assessment of whether these markers were abnormally elevated relative to normal circulating concentrations. The concentration of TGF-β1 was measured from serum rather than plasma. The clotting process during serum separation has been associated with a large release of TGF-β1 from the platelets [55]. Although no correlation was shown between the platelet count and TGF-β1 concentration, the impact of platelet TGF-β1 release during clotting was unknown in this cohort, and a comparison with plasma concentrations may have proved useful. The balance of collagen synthesis and degradation determines the accumulation of myocardial fibrosis. In this study, the collagen degradation markers (collagen I carboxy-terminal propeptide, metalloproteinases, and their tissue inhibitors) were not measured [56]. This study represents a single snapshot of a complex and dynamic disease process where patients present at various time points in the trajectory of the disease. A single assessment of these dynamic processes and of proteins that follow temporal trends over the disease course is perhaps less useful for understanding how we can best use these biomarkers in clinical practice. Transforming growth factor-beta 1 is not a cardiac-specific inflammatory marker. While patients with selected inflammatory conditions were excluded, several other inflammatory contributions exist in the clinical setting that were not accounted for in this study. This serves as an important limitation in this study.

## 6. Conclusions

This study used ELISA and CMR to evaluate the utility of TGF-β1, PICP, and PIIINP as potential biomarkers of CMR-derived myocardial fibrosis. Serum TGF-β1 holds promise as a potential surrogate marker of diffuse interstitial fibrosis measured by T1 mapping and ECV on CMR. This is important given the powerful utility of T1 mapping in predicting mortality and other clinical outcomes, symptom onset, adverse LV remodeling, and the transition towards decompensation—the latter two, illustrated in this study. Small sample sizes and other technical considerations may have limited the ability to illustrate any meaningful relationships between the selected biomarkers and replacement fibrosis measured by LGE imaging on CMR. However, an important biological explanation was also considered for this observation. Finally, the impact of PICP and PIIINP temporality in AS may limit their utility as surrogate markers of diffuse interstitial fibrosis measured by T1 mapping, but herein lies its potential strength as a marker of disease progression over time—a feature that warrants further investigation in larger prospectively designed studies.

## Figures and Tables

**Table 1:** Baseline characteristics of the total study cohort and of two subgroups divided by global T1 relaxation time (low vs. high T1 groups).

Parameter	Total Cohort (*n* = 21)	Low T1 (*n* = 10)	High T1 (*n* = 11)	*p* Value
Age (years)	60 ± 11	63 ± 12	57 ± 10	*p* = 0.21
Sex				
Female *n* (%)	13 (62)	8 (80)	5 (45)	*p* = 0.10
Male *n* (%)	8 (38)	2 (20)	6 (55)	*p* = 0.10
Chest pain *n* (%)	11 (52)	5 (50)	6 (55)	*p* > 0.99
Dyspnoea *n* (%)	20 (95)	9 (90)	11 (100)	*p* = 0.28
Orthopnoea *n* (%)	9 (43)	4 (40)	5 (45)	*p* = 0.80
Pre-syncope *n* (%)	7 (33)	4 (40)	3 (27)	*p* = 0.54
Syncope *n* (%)	6 (29)	3 (30)	3 (27)	*p* = 0.89
Fatigue *n* (%)	14 (67)	8 (80)	6 (55)	*p* = 0.22
Hypertension *n* (%)	13 (62)	8 (80)	5 (45)	*p* = 0.10
Diabetes mellitus *n* (%)	6 (29)	2 (20)	4 (36)	*p* = 0.41
Dyslipidaemia *n* (%)	9 (43)	4 (40)	5 (45)	*p* = 0.80
Smoking status				
Current use *n* (%)	4 (19)	2 (20)	2 (18)	*p* = 0.92
Past user *n* (%)	2 (10)	2 (20)	0 (0)	*p* = 0.14
Heart failure *n* (%)	9 (43)	3 (30)	6 (55)	*p* = 0.26
Heart rate (beats per minute)	75 ± 15	72 ± 15	77 ± 15	*p* = 0.49
Resting SBP (mmHg)	128 ± 20	128 ± 16	128 ± 23	*p* = 0.99
Resting DBP (mmHg)	75 ± 13	75 ± 15	75 ± 11	*p* = 0.90
Body mass index (kg/m^2^)	29.07 ± 6.08	29.38 ± 6.56	28.79 ± 5.91	*p* = 0.83
Mean AVA (cm^2^)	0.65 ± 0.21	0.58 ± 0.13	0.72 ± 0.26	*p* = 0.15
Mean gradient (mmHg)	50 (41–56)	53 (46–57)	45 (40–53)	*p* = 0.13
Peak gradient (mmHg)	75 (66–90)	83 (72–92)	72 (59–96)	*p* = 0.40

Low T1 group (mean T1 relaxation time = 1023 ± 16 ms) versus high T1 group (1056 ± 18 ms). Data presented as means ± standard deviation for normally distributed data, median (interquartile range) for non-normally distributed data, and absolute number (percentage) for categorical data. The *p* values represent differences between the low vs. high T1 group and *p* values < 0.05 were considered significant. Incidental amyloidosis excluded on CMR. SBP, systolic blood pressure; DBP, diastolic blood pressure; AVA, aortic valve area.

**Table 2:** Comparison of LV remodeling, functional and tissue characteristics in patients with low versus high T1 relaxation time.

Parameter	Total Cohort (*n* = 21)	Low T1 (*n* = 10)	High T1 (*n* = 11)	*p* Value
Global native T1 time (ms)	1040 ± 24	1023 ± 16	1056 ± 18	***p* < 0.01**
LVEDV (mL)	173.8 (138.5–229.2)	156.8 ± 40.9	228.0 ± 79.4	***p* = 0.02**
LVEDVi (mL/m^2^)	95.6 (69.1–121.7)	83.2 ± 29.6	116.8 ± 39.5	***p* = 0.04**
LVESV (mL)	80.0 (61.3–159.1)	75.7 ± 35.1	151.0 ± 83.5	***p* = 0.02**
LVESVi (mL/m^2^)	44.0 (28.7–93.1)	36.9 (26.1–46.6)	73.6 (33.2–124.8)	*p* = 0.05
LVSV (mL)	78.9 ± 26.3	81.1 ± 29.6	77.0 ± 24.1	*p* = 0.73
LVSVi (mL/m^2^)	40.5 ± 12.9	41.0 (30.8–51.6)	33.8 (28.5–54.4)	*p* = 0.70
LVM (g)	154.5 ± 48.6	128.8 ± 32.8	177.9 ± 50.0	***p* = 0.02**
LVMi (g/m^2^)	79.4 ± 23.1	67.0 ± 16.9	91.0 ± 22.8	***p* = 0.01**
M/V	0.8 ± 0.2	0.8 ± 0.2	0.8 ± 0.2	*p* = 0.99
LVEF (%)	41 (30–60)	55 (38–69)	30 (21–56)	***p* = 0.04**
MAPSE	7 ± 4	9 ± 4	6 ± 3	*p* = 0.14
ESWS (×10^3^ dynes/cm^2^)	210.4 ± 105.7	196.5 ± 94.8	223.1 ± 117.9	*p* = 0.57
LVGFI	28.4 ± 11.1	33.9 ± 9.0	23.4 ± 10.7	***p* = 0.02**
LV phenotype *				
Normal	12 (57)	9 (90)	3 (27)	***p* = 0.004**
Concentric hypertrophy	1 (5)	0 (0)	1 (9)	*p* = 0.33
Decompensation	8 (38)	1 (10)	7 (64)	***p* = 0.01**
Global T2 time (ms)	48 ± 2	49 ± 2	48 ± 2	*p* = 0.30
ECV (%)	25 ± 3	24 ± 3	26 ± 3	*p* = 0.07
LGE mass (g) (visual)	3.2 (0.1–5.2)	3.0 (0.1–5.1)	3.2 (0.1–5.9)	*p* = 0.99
LGE % (visual)	3.5 (0.1–6.3)	3.9 (0.1–7.9)	3.5 (0.1–5.1)	*p* = 0.56
LGE mass (g) (3SD)	10.7 (5.2–14.2)	11.5 (5.9–13.1)	7.2 (4.7–20.7)	*p* = 0.76
LGE % (3SD)	11.0 (6.6–19.2)	13.2 (9.9–21.6)	7.7 (4.9–18.9)	*p* = 0.17

Data presented as means ± standard deviation for normally distributed data and median (interquartile range) for non-normally distributed data. The *p* values represent differences between the low vs. high T1 group. Statistically significant *p* values (*p* < 0.05) are denoted by bold text. Volumes indexed for body surface area denoted by the letter “i”. *—Phenotype adapted from Dweck et al. [38]. LVEDV, left ventricular end-diastolic volume; LVESV, left ventricular end-systolic volume; LVSV, left ventricular stroke volume; LVM, left ventricular mass; M/V, mass to volume ratio; LVEF, left ventricular ejection fraction; MAPSE, mitral annular plane systolic excursion; ESWS, end-systolic wall stress; LVGFI, left ventricular global function index; ECV, extracellular volume; LGE, late gadolinium enhancement; 3SD, three-standard deviations.

## Data Availability

The dataset analyzed for this publication is available upon reasonable request to the corresponding author.

## Abbreviations

AS	aortic stenosis
LVEF	left ventricular ejection fraction
LV	left ventricle/left ventricular
BNP	B-type natriuretic peptide
NT-pro BNP	N-terminal pro-B-type natriuretic peptide (NT-pro BNP)
CMR	cardiovascular magnetic resonance
LGE	late gadolinium enhancement
TGF-β1	transforming growth factor beta-1
PICP	procollagen I carboxy-terminal propeptide
PIIINP	procollagen III amino-terminal propeptide
AVA	aortic valve area
ECV	extracellular volume
LVEDV	left ventricular end-diastolic volume
LVESV	left ventricular end-systolic volume
LVSV	left ventricular stroke volume
LVM	left ventricular mass
ESWS	end-systolic wall stress
LVGFI	left ventricular global function index
ELISA	enzyme-linked immunosorbent assay
SBP	systolic blood pressure
DBP	diastolic blood pressure
MAPSE	mitral annular plane systolic excursion
